# Host dysbiosis negatively impacts IL-9-producing T-cell differentiation and antitumour immunity

**DOI:** 10.1038/s41416-020-0915-6

**Published:** 2020-06-05

**Authors:** Rafael Ribeiro Almeida, Raquel de Souza Vieira, Angela Castoldi, Fernanda Fernandes Terra, Amanda Campelo L. Melo, Maria Cecília Campos Canesso, Luísa Lemos, Marcella Cipelli, Nisha Rana, Meire Ioshie Hiyane, Erika L. Pearce, Flaviano dos Santos Martins, Ana Maria Caetano de Faria, Niels Olsen Saraiva Câmara

**Affiliations:** 1 Laboratory of Transplantation Immunobiology, Department of Immunology, Institute of Biomedical Sciences, University of São Paulo, São Paulo, Brazil; 2 Laboratory of Immunology, Heart Institute (INCOR), University of São Paulo, São Paulo, Brazil; 3 Department of Biochemistry and Immunology, Biological Science Institute, Federal University of Minas Gerais, Belo Horizonte, Brazil; 4 Department of Immunometabolism, Max Planck Institute of Epigenetics and Immunobiology, Freiburg im Breisgau, Germany; 5 Department of Microbiology, Biological Science Institute, Federal University of Minas Gerais, Belo Horizonte, Brazil

**Keywords:** Immunosurveillance, Microbiome

## Abstract

**Background:**

Host–microbiota interactions shape T-cell differentiation and promote tumour immunity. Although IL-9-producing T cells have been described as potent antitumour effectors, their role in microbiota-mediated tumour control remains unclear.

**Methods:**

We analysed the impact of the intestinal microbiota on the differentiation of colonic lamina propria IL-9-producing T cells in germ-free and dysbiotic mice. Systemic effects of the intestinal microbiota on IL-9-producing T cells and the antitumour role of IL-9 were analysed in a model of melanoma-challenged dysbiotic mice.

**Results:**

We show that germ-free mice have lower frequency of colonic lamina propria IL-9-producing T cells when compared with conventional mice, and that intestinal microbiota reconstitution restores cell frequencies. Long-term antibiotic treatment promotes host dysbiosis, diminishes intestinal IL-4 and TGF-β gene expression, decreases the frequency of colonic lamina propria IL-9-producing T cells, increases the susceptibility to tumour development and reduces the frequency of IL-9-producing T cells in the tumour microenvironment. Faecal transplant restores intestinal microbiota diversity, and the frequency of IL-9-producing T cells in the lungs of dysbiotic animals, restraining tumour burden. Finally, recombinant IL-9 injection enhances tumour control in dysbiotic mice.

**Conclusions:**

Host–microbiota interactions are required for adequate differentiation and antitumour function of IL-9-producing T cells.

## Background

Multicellular organisms coexist with a symbiotic commensal microbiota. A complex community of bacteria, fungi, protozoa and viruses colonises humans, especially the gut, and plays a fundamental role in controlling host physiology.^1^ The crosstalk between the intestinal microbiota and the immune system allows tolerance of commensal bacteria and oral food antigens, and also enables recognition and control of opportunistic bacteria, preventing host invasion and infection. Along with local effects, the intestinal microbiota also have a broad range, shaping innate and adaptive immunities at multiple levels.^2^

Studies performed in germ-free (GF) mice, raised in the absence of live microbes, have revealed that ablation of the microbiota results in severe defects in the immune system, leading to altered immunoglobulin A (IgA) secretion, absent intestinal mucous layer, small Peyer’s patches and underdeveloped mesenteric lymph nodes (mLNs).^3,4^ On the other hand, different experimental approaches have demonstrated that manipulation of the intestinal microbiota may result in local and systemic immune restoration. For instance, full intestinal microbial colonisation of GF mice was shown to drive expansion of peritoneal IL-17-producing γδ T cells,^5^ while *Bacteroides fragilis* colonisation corrected systemic T-cell deficiencies and Th1/Th2 imbalances in a PSA-dependent mechanism.^6^ Segmented filamentous bacteria (SFB) and microbiota-derived ATP were shown to restore lamina propria Th17 cells in GF animals,^7–9^ while colonisation with *B. fragilis* and indigenous *Clostridium* species induced colonic regulatory T-cell (Treg) differentiation via PSA/TLR2 engagement, and by promoting a transforming growth factor-β (TGF-β)-rich environment.^10–12^

The intestinal microbiota composition is fine-tuned and subjected to different perturbations that may result in a less-diverse and less-stable architecture known as dysbiosis, which is frequently associated with impaired local and systemic immune responses.^13–15^ GF mice colonised with intestinal microbiota from animals with inflammation-associated colorectal cancer were shown to be more susceptible to tumorigenesis,^16^ and administration of *Fusobacterium nucleatum* was shown to promote a proinflammatory microenvironment, and to potentiate intestinal tumorigenesis in specific pathogen-free (SPF) mice.^17^ Systemically, it was demonstrated that a dramatic reduction of gut commensals via broad-spectrum antibiotic treatment resulted in increased susceptibility to melanoma and Lewis carcinoma development in mice, negatively impacting IL-17-producing γδ T cells.^18^ More recently, antibiotic-induced dysbiosis was shown to enhance distal tumour progression by altering host cytokine levels and endothelial adhesion molecules, resulting in a defective recruitment of effector CD8 + T cells into the tumour microenvironment.^19^ Many other studies have also indicated a fundamental role of the microbiota in regulating responses to cancer therapies, such as CpG-based immunotherapy, platinum-based chemotherapy and checkpoint inhibitors anti-CTLA4 and anti-PD1/PDL-1.^20–23^

Although host–microbiota interactions have been described to regulate the differentiation and function of many effector and regulatory T cells, contributing to homoeostasis and tumour immunity, no study has addressed whether these interactions would have an impact on IL-9-producing CD4 + (Th9) and CD8 + (Tc9) T cells. Th9 and Tc9 cells share developmental pathways with both Th2 and Treg cells, being differentiated in response to IL-4 and TGF-β.^24^ These cells are mainly characterised by IL-9 production, and also by the expression of the transcription factors PU.1, STAT6 and IRF4.^25–29^ While Th9 cells were shown to be more efficient than Th1 and Th17 cells in preventing tumour progression, to promote IL-9-dependent antitumour immunity, and to have direct cytotoxic effects on tumour cells,^30–32^ Tc9 cells were shown to be superior effectors than type-I cytotoxic Tc1 cells for adoptive cancer immunotherapy, and to also depend on IL-9 for their antitumour response.^29,33^ Here, we investigated the role of the intestinal microbiota in promoting the development of lamina propria Th9 and Tc9 cells by reconstituting germ-free mice with faecal microbiota from conventional mice. We also performed long-term antibiotic treatment in specific pathogen-free mice to evaluate the impact of host dysbiosis on IL-9-producing T cells in the colonic lamina propria, and in the context of extraintestinal tumour immunity. Taken together, our results indicate that host–microbiota interactions drive differentiation and antitumour function of IL-9-producing T cells.

## Methods

### Germ-free mice and conventionalisation

Six to 8-week-old male conventional (CV), germ-free (GF) and germ-free conventionalised (CVN) Swiss mice were used for analysis of colonic lamina propria cells at the Federal University of Minas Gerais in accordance with the National Institutes of Health guide for the care and use of Laboratory animals and the guidelines of the Institutional Ethics Committee. Animals were housed in groups of up to five per cage in a light- and temperature-controlled room (12-h light/dark cycles, 21 ± 2 °C) with free access to food and water. Conventionalisation was achieved by oral gavage of PBS-homogenised faecal samples from the large intestine of CV mice to GF mice, which were also left in co-housing with CV mice for 21 days prior to immunological analysis. Animals were euthanised with an overdose of isoflurane, and experiments were performed after trained personnel recognised cessation of vital signs. Three independent experiments were performed with 6–8 animals per group. A completed ARRIVE guidelines is provided as Supplementary Information (Checklist S1).

### Antibiotic treatment and faecal microbiota transplantation

Four to 5-week-old male SPF C57BL/6 mice received an antibiotic cocktail twice a day by gavage for 4 weeks, as previously described.^34^ Animals were housed in groups of up to five per cage in a light- and temperature-controlled room (12-h light/dark cycles, 21 ± 2 °C) with free access to food and water. To achieve microbiota recolonisation, antibiotic treatment was interrupted for 2 days, and PBS-homogenised faecal samples were given by gavage through 4 consecutive days. Mice were then left untreated with antibiotics for an additional 15 days, and injected with B16F10 cells. Antibiotic-treated mice injected with B16F10 cells were kept under treatment throughout tumour development. Experiments were performed in accordance with the National Institutes of Health guide for the care and use of Laboratory animals, and with the guidelines of the Ethics committee of the University of São Paulo, under protocol number 2015/006. Animals were euthanised with an overdose of isoflurane, and experiments were performed after trained personnel recognised cessation of vital signs. Three independent experiments were performed with 8–10 animals per group.

### Microbiota analysis

Microbiota depletion was evaluated by culturing faecal samples in fluid thioglycolate media (Neogen, Lansing, MI, USA) for 3 days in a microbiological incubator at 37 °C and 180 rpm. Alternatively, bacterial load in faecal samples was determined by 16S RNA quantification, as previously described.^34^ For metagenomic analysis, DNA was extracted from faecal samples using the QIAamp DNA Mini Stool kit (Qiagen, Germantown, MD, USA) and quantified. The 16S rRNA gene amplification and sequencing were performed according to Illumina MiSeq 16S Metagenomic Sequencing Library Preparation protocol at CEFAP/ICB-USP. Amplicons were multiplexed using the Nextera XT Index kit. The quality of libraries was assessed using Bioanalyzer (Agilent, Santa Clara, CA, USA) and quantified using Qubit (Thermo Fisher, Waltham, MA, USA). Sequence analysis was performed using the Qiime package version 1.9.1 and Qiime2.

### B16F10 culture and injection

B16F10 cells (ATCC) were cultured in RPMI (Thermo Fisher) with 10% FBS (Thermo Fisher) at 37 °C and 5% CO_2_. C57BL6 mice were injected with 5 × 10^4^ B16F10 cells through the tail vein, and tumour foci number was determined in the lungs 14 days after injection. Animals were euthanised as previously described. Three independent experiments were performed with 10–12 animals per group.

### IL-9 treatment

Antibiotic-treated mice were intraperitoneally injected either with 250 ng of recombinant IL-9 (R&D Systems, Minneapolis, MN, USA) diluted in 200 µl of PBS or PBS alone at the day of B16F10 cells’ injection and every other day throughout tumour development. Animals were euthanised as previously described. Two independent experiments were performed with 6–8 animals per group.

### Isolation of lamina propria cells

Colonic lamina propria cells were isolated as detailed.^35^ Briefly, animals were euthanised as previously described, and the large intestine was dissected, longitudinally opened, washed with PBS and cut into small pieces. Tissue fragments were placed in Petri dishes and washed three times in calcium- and magnesium-free HBSS containing 1 mM dl-dithiothreitol (DTT, Sigma-Aldrich, Irvine, UK) for 30 min. Supernatants were discarded. After that, tissue fragments were incubated with 100 µl/mL of collagenase II (Sigma-Aldrich) for 60 min at 37 °C on a shaker. Supernatants were passed through a 70-µm cell strainer (Falcon, Corning, NY, USA), and then resuspended in R-10 medium (RPMI with 10% FBS, 2 mM L-glutamine (Thermo Fisher), 1 mM sodium pyruvate (Thermo Fisher), 1% non-essential amino acids (Thermo Fisher), 1% Pen/Strep, 1% vitamin solution (Thermo Fisher) and 5 × 10^−5^ M 2β-mercaptoetanol) for further analysis.

### Isolation of lung cells

Mice were euthanised as previously described, and lungs collected, washed in ice-cold PBS, cut into small pieces and incubated in R-10 medium with 0.5 mg/ml collagenase IV (Thermo Fisher) and 30 μg/ml DNAse at 37 °C for 45 min and 180 rpm. Digested tissues were passed through 100-μm cell strainers and centrifuged. Pellets were resuspended in 1 ml of ACK buffer (Thermo Fisher) for 2 min, centrifuged and resuspended in R-10 medium for further analysis.

### Flow cytometry

Cells from lamina propria and lungs were stimulated with PMA (Sigma-Aldrich) 50 ng/ml, Ionomycin (Sigma-Aldrich) 500 ng/ml and Brefeldin A (Biolegend, San Diego, CA, USA) 5 µg/ml for 4 h at 37 °C and 5% CO_2_. T-cell surface staining was performed for 30 min at 4 °C using the following antibodies diluted in PBS: anti-CD45 PercP (BD, Franklin Lakes, NJ, USA), anti-CD4 APCCy7 (Biolegend) and anti-CD8 FITC (Biolegend). Cells were then fixed and permeabilised using the Cytofix/Cytoperm kit (BD). Intracellular staining was performed for 30 min at 4 °C with the following antibodies diluted in Perm/wash buffer (BD): anti-CD3 APC (BD), anti-IL-9 PE (Biolegend) and anti-IFN-γ PECy7 (Biolegend). Lung cells were also stained for mast cell detection with anti-CD45 PercP and anti-CD117 APC (Biolegend). Samples were acquired using a FACS Canto II (BD) and analysed using FlowJo software (version 9.0.2, Tree Star).

### Real-time qPCR

The total RNA was extracted from colon samples using Trizol reagent (Thermo Fisher), and cDNA synthesised with M-MLV reverse transcriptase (Promega, Madison, WI, USA) according to the manufacturer’s instructions. Real-time qPCR was performed using SyBr green master mix (Thermo Fisher) and a QuantStudio 12k (Thermo Fisher) with the following parameters: 95 °C for 15 min, 40 cycles of 94 °C for 15 s, 58 °C for 30 s and 72 °C for 30 s. TGF-β, IL-4 and HPRT primers were purchased from Sigma-Aldrich.

### Statistical analysis

Statistical analysis was carried out with Graph Pad Prism 6.0 Software.

## Results

### Host microbiota play a key role in the differentiation of IL-9-producing T cells

**Fig. 1:**
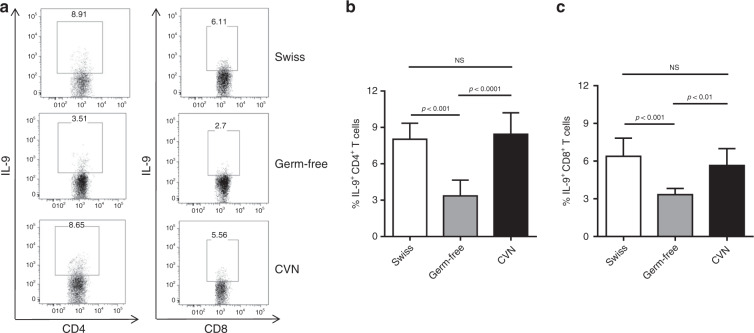
Host microbiota play a key role in the development of intestinal IL-9-producing T cells. Colonic lamina propria cells from conventional Swiss, germ-free or germ-free conventionalised (CNV) mice were extracted and stimulated with PMA (50 ng/ml), ionomycin (500 ng/ml) and Brefeldin A (5 μg/ml) for 4 h at 37 °C and 5% CO_2_, and analysed by flow cytometry (**a**) to determine the frequency of IL-9-producing CD4 + (**b**) and CD8 + (**c**) T cells. Data are shown as mean ± SD. One-way ANOVA followed by Tukey’s multiple-comparison test was used for statistical analysis.

**Fig. 2:**
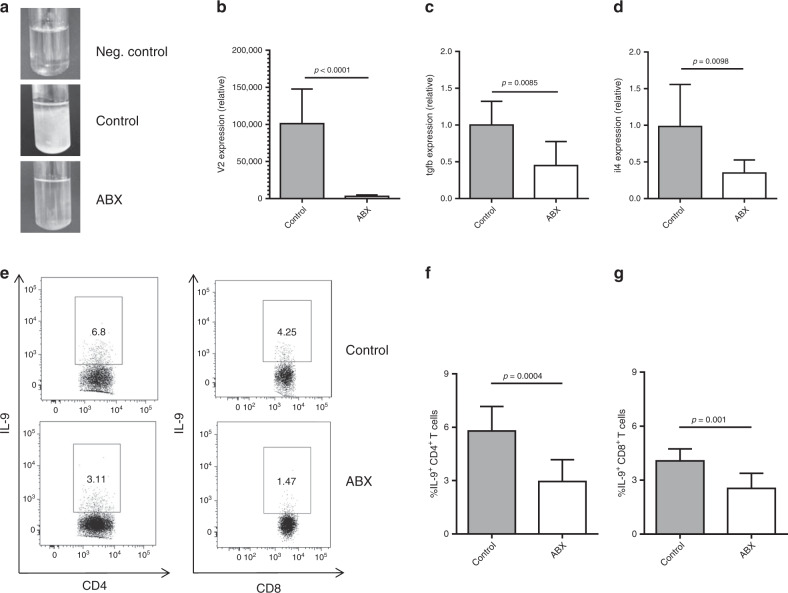
Host dysbiosis negatively impacts intestinal IL-9-producing T cells. Specific pathogen-free C57BL/6 mice received an antibiotic cocktail twice a day by gavage for 4 weeks (ABX), or were left untreated (control). Microbiota depletion in faecal pellets was evaluated by thioglycolate culture (**a**) and bacterial 16 S V2 DNA load (**b**) at the end of treatment. The total RNA was extracted from the colon, and relative expression of TGF-β (**c**) and IL-4 (**d**) was determined by real-time qPCR. Colonic lamina propria cells from control and ABX mice were extracted and stimulated with PMA (50 ng/ml), ionomycin (500 ng/ml) and Brefeldin A (5 μg/ml) for 4 h at 37 °C and 5% CO_2_, and analysed by flow cytometry (**e**) to determine the frequency of IL-9-producing CD4 + (**f**) and CD8 + (**g**) T cells. Data are shown as mean ± SD. Unpaired Student’s *t* test was used for statistical analysis.

It is well-established that host–microbiota interaction promotes the differentiation of many T-cell subsets.^5,8,11^ Therefore, we asked whether the differentiation of IL-9-producing T cells would also depend on it. To answer this question, we first evaluated colonic lamina propria of germ-free Swiss mice and found that the frequency of Th9 and Tc9 cells was significantly lower when compared with controls (Fig. 1a–c), while conventionalisation of germ-free mice restored the frequency of both cells (Fig. 1a–c). In addition, we performed a long-term antibiotic (ABX) treatment to deplete the microbiota of C57BL6 mice, and observed a drastic reduction in faecal bacterial load (Fig. 2a, b). No significant body weight loss was observed in ABX-treated animals (data not shown). We found that TGF-β and IL-4 gene expression, key cytokines for Th9 and Tc9 differentiation, was significantly impaired in colons of ABX-treated animals (Fig. 2c, d, respectively). Flow cytometry analysis showed a significantly lower frequency of Th9 and Tc9 cells in the colonic lamina propria of ABX-treated animals (Fig. 2e–g). Taken together, these results indicate that the host microbiota play a key role in the differentiation of IL-9-producing T cells, and that the mechanism may rely on TGF-β and IL-4 expression.

### Antibiotic treatment negatively modulates IL-9-producing T cells in tumour microenvironment and enhances tumour development

**Fig. 3:**
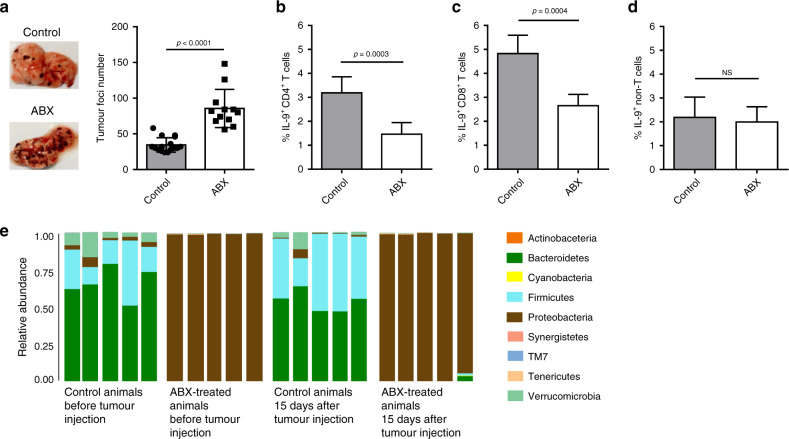
Antibiotic-treated mice have a higher number of tumour foci and lower lung-infiltrating IL-9-producing T cells. Antibiotic-treated (ABX) and -untreated (control) C57BL/6 mice were intravenously injected with 5 × 10^4^ B16F10 cells. Tumour foci number in the lungs was determined 14 days after injection (**a**). Lungs were then digested, and cells stimulated with PMA (50 ng/ml), ionomycin (500 ng/ml) and Brefeldin A (5 μg/ml) for 4 h at 37 °C and 5% CO_2_. The frequency of IL-9-producing CD4 + (**b**) and CD8 + (**c**) T cells and IL-9-producing non-T cells (**d**) was evaluated by flow cytometry. Metagenomic analysis of the gut microbiota was performed in faecal samples collected at the day of and 2 weeks after injection of B16F10 cells (**e**). Mice from the ABX group were kept under treatment throughout the experiment. Data are shown as mean ± SD. Unpaired Student’s *t* test was used for statistical analysis.

Given the fact that the host microbiota were important for the differentiation of both Th9 and Tc9 cells in the colonic lamina propria, we next sought to evaluate whether a long-term antibiotic treatment would impair IL-9-producing T cells in an extraintestinal tumour model. To address this question, we injected B16F10 cells in ABX-treated and -untreated animals through the tail vein in order to promote tumour growth in the lungs. We demonstrated that ABX-treated mice were more susceptible to tumour development than controls, as shown by a higher number of lung foci (Fig. 3a). We observed a significantly lower frequency of Th9 and Tc9 cells (Fig. 3b, c, respectively) in the lungs of ABX-treated animals. In contrast, no difference in the frequency of IL-9-producing non-T cells was found (Fig. 3d). Metagenomic analysis showed that antibiotic treatment reduced microbiota diversity before injection of B16F10 cells, which was maintained throughout tumour development (Fig. 3e). Overall, these data indicate that antibiotic-mediated dysbiosis negatively modulates IL-9-producing T cells in the tumour microenvironment, and that antibiotic-treated animals are more susceptible to tumour development.

### Faecal transplant restores IL-9-producing T cells in the tumour microenvironment and protects mice from tumour development

**Fig. 4:**
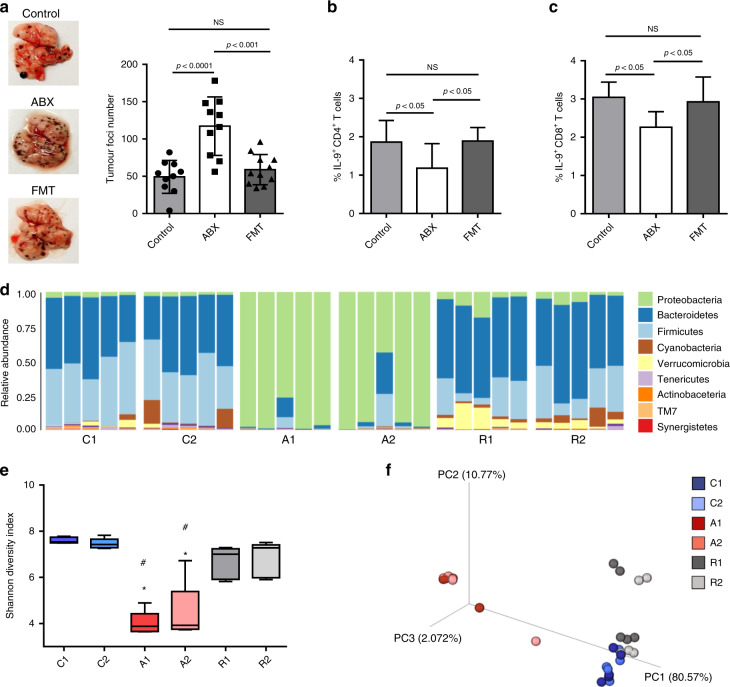
Faecal microbiota transplant restores lung-infiltrating IL-9-producing T cells and protects antibiotic-treated mice from tumour development. C57BL/6 mice received an antibiotic cocktail twice a day by gavage for 4 weeks (ABX), or were left untreated (control). After 4 weeks of treatment, a group of ABX-treated mice received faecal microbiota transplant (FMT), while the other group was kept under treatment. All mice were intravenously injected with 5 × 10^4^ B16F10 cells, and tumour foci number in the lungs was determined 14 days after injection (**a**). Lungs were then digested, and cells stimulated with PMA (50 ng/ml), ionomycin (500 ng/ml) and Brefeldin A (5 μg/ml) for 4 h at 37 °C and 5% CO_2_. The frequency of IL-9-producing CD4 + (**b**) and CD8 + (**c**) T cells was evaluated by flow cytometry. Metagenomic analysis of the gut microbiota was performed in faecal samples collected at the day of and 2 weeks after injection of B16F10 cells (**d**). Microbiota alpha-diversity was determined by the Shannon index (**e**). Principal coordinate analysis (PCoA) based on weighted Unifrac metric of faecal microbiota among all samples was also performed (**f**). Data are shown as mean ± SD. One-way ANOVA followed by Tukey’s multiple-comparison test and Kruskal–Wallis test was used for statistical analysis. Control animals before (C1) and 15 days after tumour injection (C2). ABX-treated animals before (A1) and 15 days after tumour injection (A2). Microbiota-reconstituted animals before (R1) and 15 days after tumour injection (R2). *p = 0.009 compared with Groups 1 and 2. ^#^*p* = 0.009 compared with Groups 5 and 6.

To confirm the role of the host microbiota in modulating Th9 and Tc9 cells in the tumour microenvironment, PBS-homogenised faecal samples from control animals were orally given to ABX-treated mice to re-establish microbiota composition before injection of B16F10 cells. We found that faecal transplants significantly impaired tumour burden in the lungs, maintaining the number of foci close to that observed in control animals (Fig. 4a). Flow cytometry analysis revealed that the frequency of both IL-9-producing CD4 + and CD8 + T cells in the lungs of faecal-transplanted animals was restored to that observed in control animals (Fig. 4b, c, respectively). Microbiota recolonisation was confirmed by metagenomic analysis of faecal samples (Fig. 4d), and we observed that antibiotic treatment significantly reduced microbiota diversity, while faecal transplant restored it (Fig. 4e). Control and faecal-transplanted animals showed similar microbiota profiles, which were different from ABX-treated animals (Fig. 4f). These results reinforced our data suggesting that the host microbiota have an important role in both modulating the frequency of Th9 and Tc9 cells into the tumour microenvironment, and contributing to protection against tumour development.

### IL-9 treatment improves tumour control in dysbiotic animals

**Fig. 5:**
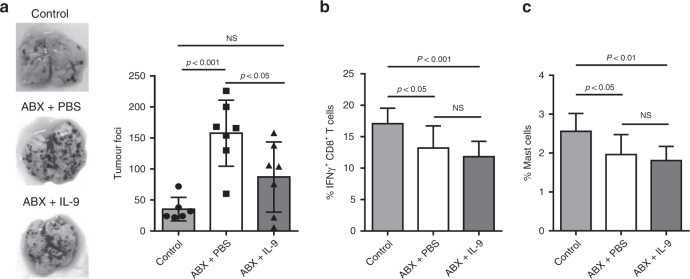
IL-9 treatment improves tumour control in dysbiotic animals. C57BL/6 mice received an antibiotic cocktail twice a day by gavage for 4 weeks (ABX) or were left untreated (control). All mice were intravenously injected with 5 × 10^4^ B16F10 cells. ABX-treated mice were intraperitoneally injected either with IL-9 or PBS every other day throughout tumour development. Tumour foci number in the lungs was determined 14 days after injection (**a**). Lungs were then digested, and cells stimulated with PMA (50 ng/ml), ionomycin (500 ng/ml) and Brefeldin A (5 μg/ml) for 4 h at 37 °C and 5% CO_2_. The frequency of IFN-γ-producing CD8 + T cells (**b**) and mast cells (**c**) was evaluated by flow cytometry. Data are shown as mean ± SD. One-way ANOVA followed by Tukey’s multiple-comparison test was used for statistical analysis.

The previous reports showing anti-tumoural properties of Th9 and Tc9 cells^29–31^ allied to our results demonstrating no difference in the frequency of IL-9-producing non-T cells in the lungs of ABX-treated mice raised the question whether IL-9 treatment would be sufficient to improve tumour control in those animals as compensation for the lower number of Th9 and Tc9 cells. We therefore injected either recombinant IL-9 or PBS in ABX-treated mice throughout tumour development, and found enhanced tumour control in IL-9-treated animals, as shown by a lower number of lung foci compared with animals treated with PBS (Fig. 5a). We also evaluated cells that have been described as important players in Th9-mediated antitumour immunity,^30,31^ and observed that ABX-treated animals presented lower frequency of IFN-γ-producing CD8 + T cells (Fig. 5b) and mast cells (Fig. 5c) in the lungs, which was not improved upon IL-9 treatment (Fig. 5b, c). Therefore, these data indicate that IL-9 contributes to microbiota-mediated control of tumours by a mechanism that may not rely on recruitment of IFN-γ-producing CD8 + T cells and mast cells into tumour microenvironment.

## Discussion

In this study, we demonstrated that the host microbiota play a key role in the differentiation of Th9 and Tc9 cells by showing that germ-free mice have a lower frequency of both cells in the colonic lamina propria when compared with conventional mice, and that faecal transplant followed by co-housing is sufficient to restore cell frequencies. We demonstrated that long-term antibiotic treatment promotes host dysbiosis, diminishes intestinal IL-4 and TGF-β gene expression, decreases the frequency of colonic lamina propria Th9 and Tc9 cells, increases the susceptibility to tumour development and reduces the frequency of Th9 and Tc9 cells in the tumour microenvironment. We found that faecal transplant is sufficient to restore intestinal microbiota diversity and the frequency of IL-9-producing T cells in the lungs of antibiotic-treated animals, restraining tumour burden. Finally, we demonstrated that recombinant IL-9 injection results in enhanced tumour control in dysbiotic mice.

Many studies indicate that host–microbiota interactions contribute to local and systemic T-cell differentiation and function.^5,8,11,36,37^ However, no study had addressed the impact of these interactions on IL-9-producing T cells. We have recently shown that the microbiota-derived short-chain fatty acid (SCFA) butyrate suppresses Th9 differentiation both in vitro and in vivo, impairing lung inflammation.^38^ Here, we have demonstrated that Swiss germ-free mice have significantly lower colonic lamina propria Th9 and Tc9 cells when compared with conventional animals, and that intestinal microbiota reconstitution is sufficient to restore cell frequencies. We also found that antibiotic-treated C57BL6 mice present a lower frequency of colonic lamina propria Th9 and Tc9 cells when compared with controls, indicating that host–microbiota interactions are important for the differentiation of these cells, independently of the studied model. Although our previous work and the present results seem contrasting, as the intestinal microbiota are responsible for SCFA production,^2^ we believe that a delicate balance between microorganisms and their products is necessary to fully modulate T-cell differentiation and function in different biological conditions.

A previous work has suggested that the host microbiota modulate tumour-immune surveillance in the lungs through an IL-17-producing γδT-cell-dependent mechanism. A lower number of αβ CD4 + and CD8 + T cells was also observed in the lungs of antibiotic-treated animals, although no further characterisation of T-cell subtypes and their contributions to tumour immunity was performed.^18^ Given the increasing evidence that the host microbiota and IL-9-producing T cells modulate tumour progression,^16–18,29,33,39^ we sought to evaluate whether Th9 and Tc9 cells would be among the players of microbiota-mediated protection against tumour development. Our results indicate that antibiotic-induced dysbiosis negatively impacts Th9 and Tc9 cells in the lungs of tumour-challenged mice, indicating that IL-9-producing T cells may pose a significant contribution to microbiota-mediated tumour-immune surveillance. We believe that low expression of IL-4 and TGF-β in dysbiotic animals may have contributed to the defective Th9 and Tc9 differentiation, as these cytokines have a central role in the differentiation of IL-9-producing T cells.^24,28,29^

As previously observed,^18,19^ dysbiotic mice were more susceptible to tumour development. We also demonstrated that faecal transplant was sufficient to restore intestinal microbiota diversity and lung-infiltrating Th9 and Tc9 cells, and to impair tumour burden. Although we cannot rule out a contribution of the lung microbiota in the differentiation of Th9 and Tc9 cells, our results with faecal transplant suggest that the intestinal microbiota may have had a major impact. The fact that the metronidazole function depends on anaerobic bacteria,^40^ and that neomycin/vancomycin-aerosolised mice present significant reduction rather than an increase of melanoma development in the lungs,^41^ also supports our hypothesis of a systemic effect of the intestinal microbiota on lung-infiltrating antitumour Th9 and Tc9 cells.

To further investigate the effects of Th9 and Tc9 cells in our model, we systemically injected recombinant IL-9 in dysbiotic animals, and found that it could partially protect against tumour burden. Systemic IL-17 injection has also been shown to impair melanoma development in the lungs of dysbiotic animals,^18^ suggesting that other cell types may be involved in tumour control. Although Th9 and Tc9 cells may exert direct effects on tumour cells and produce other cytokines,^29,32,42^ it is well-established that IL-9 has an important contribution to their antitumour immunity.^30,31,33^ We understand that additional studies regarding T-cell transfer are necessary to confirm the antitumour role of Th9 and Tc9 cells in dysbiotic animals, but our hypothesis is still supported by the fact that IL-9-producing non-T cells were unchanged in the lungs of dysbiotic tumour-challenged mice, indicating that T lymphocytes are probably the main source of IL-9 antitumour effects in our model.

Searching for a mechanism underlying IL-9-mediated protection against tumour burden in dysbiotic animals, we evaluated lung-infiltrating IFN-γ-producing CD8 + T cells and mast cells, and found that dysbiotic tumour-challenged animals had a lower frequency of both cells when compared with controls, although it was not improved upon IL-9 treatment. Contrasting information suggests that Th9/IL-9-protective effects rely on the recruitment of either CD8 + T cells or mast cells into the tumour microenvironment.^30,31^ Here, we found that IL-9 may promote antitumour immunity through a different mechanism when in the context of dysbiosis. For instance, recent data indicate that dysbiosis impairs TNF production, expression of endothelial adhesion molecules and the consequent recruitment of protective IFN-γ-producing CD8 + T cells into the tumour microenvironment,^19^ which could partially support our findings. It is also possible that other cell types may have exerted antitumour effects in dysbiotic animals upon IL-9 treatment, and that activation rather than recruitment of mast cells is an important factor.^43^

Dysbiosis has been associated not only with poor intrinsic antitumour immunity, but also with lack of efficacy of different therapies, particularly checkpoint inhibitors, both in animal models and human subjects.^20–22,44,45^ Therefore, improved understanding of how microbiota modulate tumour progression is urgently needed so that better clinical outcomes can be reached. Our study brings an important contribution to the field by demonstrating that dysbiosis affects important players in tumour immunity, such as IL-9-producing T cells, and suggests that these cells should be carefully considered in further studies regarding the impacts of the host microbiota on cancer immunology and therapy.

## Data Availability

The data sets generated and analysed during this study are not publicly available, but available from the corresponding author on reasonable request.
